# Role of osteogenic Dickkopf-1 in bone remodeling and bone healing in mice with type I diabetes mellitus

**DOI:** 10.1038/s41598-021-81543-7

**Published:** 2021-01-21

**Authors:** Nick Hildebrandt, Juliane Colditz, Caio Dutra, Paula Goes, Juliane Salbach-Hirsch, Sylvia Thiele, Lorenz C. Hofbauer, Martina Rauner

**Affiliations:** 1 Department of Medicine III and Center for Healthy Aging, Medical Faculty, Technische Universität Dresden, Fetscherstr. 74, 01307 Dresden, Germany; 2 Post-Graduation Program in Morphological Science, Department of Morphology, School of Medicine, Federal University of Ceará, Fortaleza, CE Brazil; 3 Department of Pathology and Legal Medicine, School of Medicine, Federal University of Ceará, Fortaleza, CE Brazil; 4 Present Address: Rudolf Schönheimer Institute of Biochemistry, Medical Faculty, University of Leipzig, Johannisallee 30, 04103 Leipzig, Germany

**Keywords:** Bone, Disease model, Endocrine system and metabolic diseases, Morphogen signalling

## Abstract

Type 1 diabetes mellitus (T1DM) is associated with low bone mass and a higher risk for fractures. Dickkopf-1 (Dkk1), which inhibits Wnt signaling, osteoblast function, and bone formation, has been found to be increased in the serum of patients with T1DM. Here, we investigated the functional role of Dkk1 in T1DM-induced bone loss in mice. T1DM was induced in 10-week-old male mice with Dkk1-deficiency in late osteoblasts/osteocytes (Dkk1^f/f^;Dmp1-Cre, cKO) and littermate control mice by 5 subsequent injections of streptozotocin (40 mg/kg). Age-matched, non-diabetic control groups received citrate buffer instead. At week 12, calvarial defects were created in subgroups of each cohort. After a total of 16 weeks, weight, fat, the femoral bone phenotype and the area of the bone defect were analyzed using µCT and dynamic histomorphometry. During the experiment, diabetic WT and cKO mice did not gain body weight compared to control mice. Further they lost their perigonadal and subcutaneous fat pads. Diabetic mice had highly elevated serum glucose levels and impaired glucose tolerance, regardless of their Dkk1 levels. T1DM led to a 36% decrease in trabecular bone volume in Cre− negative control animals, whereas Dkk1 cKO mice only lost 16%. Of note, Dkk1 cKO mice were completely protected from T1DM-induced cortical bone loss. T1DM suppressed the bone formation rate, the number of osteoblasts at trabecular bone, serum levels of P1NP and bone defect healing in both, Dkk1-deficient and sufficient, mice. This may be explained by increased serum sclerostin levels in both genotypes and the strict dependence on bone formation for bone defect healing. In contrast, the number of osteoclasts and TRACP 5b serum levels only increased in diabetic control mice, but not in Dkk1 cKO mice. In summary, Dkk1 derived from osteogenic cells does not influence the development of T1DM but plays a crucial role in T1DM-induced bone loss in male mice by regulating osteoclast numbers.

## Introduction

Type 1 diabetes mellitus (T1DM) is an autoimmune disorder that is most often diagnosed in children and young adults^1,2^. Thus, clinical management of T1DM is required throughout life. Metabolic pathways that are altered by T1DM impose a constant damage to most tissues and organ systems. While vascular, renal, ophthalmologic, and neuronal complications are widely known, other organs such as bone have also come into focus during more recent years. Patients with T1DM face an over sixfold increased risk of hip fractures, which are frequently responsible for chronic immobilization, reduced quality of life and death^3–5^. Various studies already established the link between T1DM, osteoporosis and impaired cortical and trabecular bone microarchitecture, e.g. reduced cortical thickness, decreased volumetric bone mineral density, as well as lower trabecular bone volume and trabecular number compared to non-diabetic controls^6–8^. Multiple reasons for the resulting structural bone fragility are in discussion, for example, the reduced secretion of the bone-anabolic insulin-like growth factor 1 (IGF-1), but also the reduced concentration of insulin itself and thereby the lack of anabolic effects on bone provided by the peptide hormone^9,10^.

Further, the level of the advanced glycation end products (AGEs) is elevated, which is known to interfere with osteoblast attachment to bone matrix and to induce apoptosis in mesenchymal stem cells (MSC), thereby compromising osteoblastogenesis and subsequently osteoblast activity^11,12^. Moreover, AGEs intercalate into the collagenous bone matrix, thereby increasing the collagenous network stiffness and render bone more susceptible to fractures^13^. In addition, mechanosensing by osteocytes is reduced in diabetic mice, which may negatively impact on the regulation of the bone remodeling cycle^14^.

The Wnt signaling pathway is one of the most important pathways to regulate bone mass. It stimulates bone formation via osteoblasts and reduces osteoclastogenesis, thereby, reducing bone resorption^15,16^. Moreover, Wnt signaling promotes mesenchymal stem cell differentiation into osteoblasts rather than adipocytes^12^. Considering that T1DM inhibits bone formation^17,18^, the question arises as to whether Wnt signaling is impaired in T1DM. In fact, studies found increased serum levels of Dickkopf-1 (Dkk1) and sclerostin, both of which are inhibitors of the Wnt pathway, in animals and human patients with T1DM^19–21^. Moreover, elevated Dkk1 levels correlated with decreased bone mineral density in patients with T1DM. However, the formal proof whether Dkk1 and/or sclerostin are implicated in bone disease due to T1DM is still lacking^22^. As previously described, bone mass regulation via Dkk1 is not dependent on systemic levels, but on local Dkk1 expression, secreted by osteoblasts and osteocytes^23^. Accordingly, we assessed here if skeletal Dkk1 production is implicated in the pathogenesis of T1DM-induced bone loss. Our results show that mice lacking Dkk1 in osteoblasts and osteocytes are not fully protected from trabecular bone loss due to T1DM, but that they lose less bone than their wildtype littermates. Cortical bone loss, however, is completely abrogated by Dkk1 deficiency. In contrast, Dkk1 does not drive impaired bone defect healing in T1DM. Thus, the effects of Dkk1 in diabetic bone disease appear to be site-specific and depending on modeling vs. remodeling activities.

## Methods

### Declaration of approval for animal experiments

All experiments involving animals were approved by the Landesdirektion Sachsen, Germany and were performed in accordance with the relevant guidelines and regulations (approval number: TVV 38/2017). All experiments were performed according to ARRIVE guidelines as well.

### Mouse models

**Figure 1:**
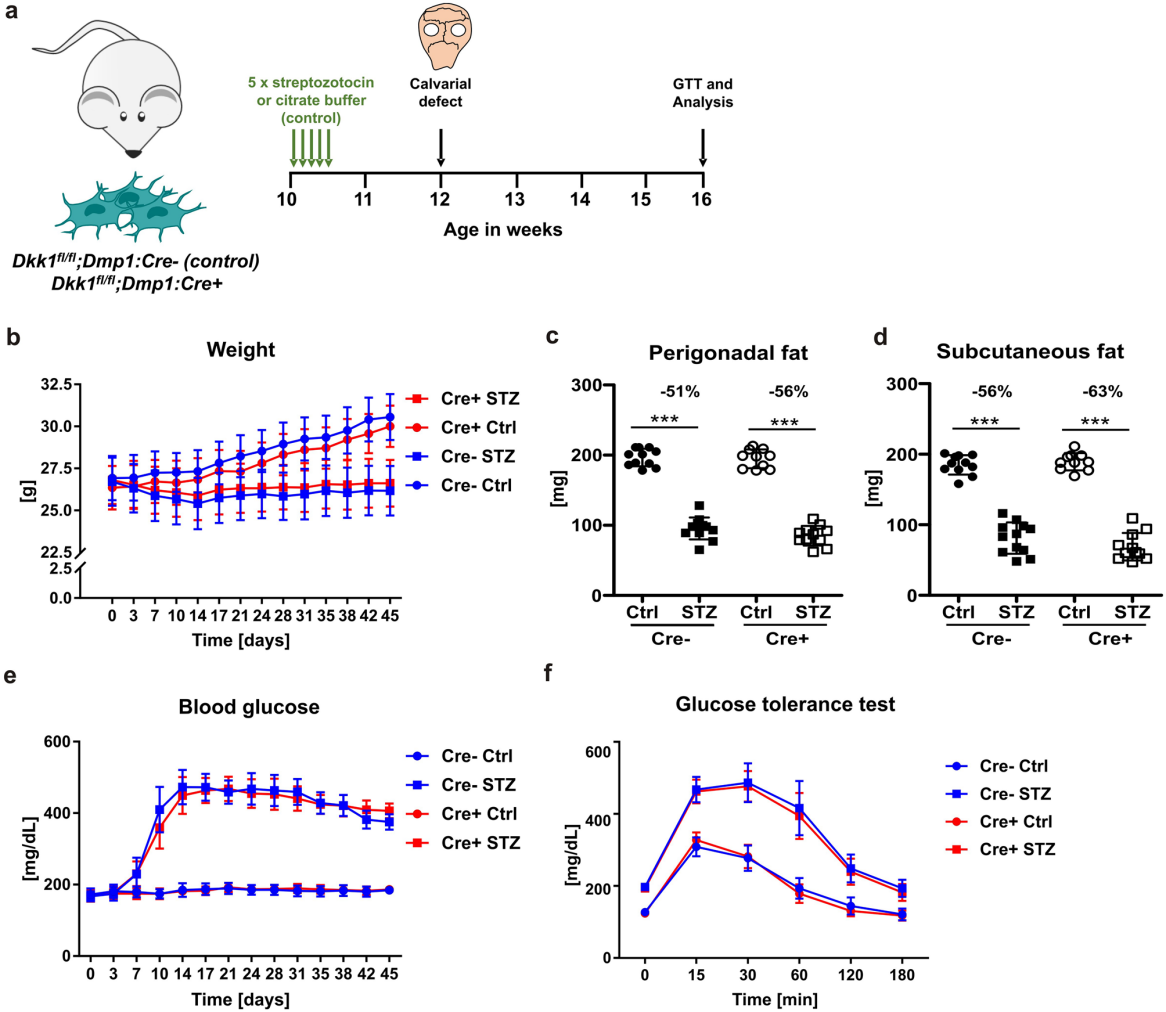
Osteogenic Dkk1 does not alter glucose homeostasis in T1DM. (**a**) Scheme of the experimental procedures. 10 week-old male Dkk1^fl/fl^;Dmp1:Cre mice and their respective Cre-negative littermates were injected 5 times with streptozotocin or citrate buffer (control group). Two weeks later the subcritical-sized calvarial defect was induced. After another four weeks, the glucose tolerance test (GTT) was conducted and mice were sacrificed afterwards for further analysis. Throughout the entire experiment, the body weight and blood glucose level of the animals was measured twice a week. (**b**) Weight was measured throughout the experiment. (**c**) Perigonadal and (**d**) subcutaneous fat pads were excised after sacrifice and weighed. (**e**) Blood glucose levels were measured throughout the experiment. (**f**) Glucose tolerance test was performed after 3 weeks of diabetes induction. All data are represented as the mean ± standard deviation or as individual dots. N = 10–12 per group. Two-way ANOVA with Bonferroni post-hoc test. ***p < 0.001 vs. Ctrl.

Dkk1^fl/fl^;Dmp1:Cre mice, which lack Dkk1 constitutively in Dmp1-expressing cells (late osteoblasts, osteocytes), were characterized previously^23^. Dkk1^fl/fl^;Dmp1:Cre− littermates were used as controls. At the age of 10 weeks, male mice were randomly divided into groups treated with streptozotocin (STZ, 40 mg/kg) and control groups receiving intraperitoneal injections of citrate buffer (Ctrl). Both agents were injected for five consecutive days using the properties of low-dose STZ therapy to induce T1DM^24^. Thus, the following cohorts were generated: *Dkk1*^*fl/fl*^*;Dmp1:Cre* Cre−* Ctrl*: n = 12, *Dkk1*^*fl/fl*^*;Dmp1:Cre*−* STZ*: n = 12, *Dkk1*^*fl/fl*^*;Dmp1:Cre*+ *Ctrl*: n = 10, *Dkk1*^*fl/fl*^*;Dmp1:Cre*+ *STZ*: n = 12. Blood glucose levels and body weight of each mouse were measured twice a week until week 16, when they were sacrificed via intraperitoneal injection of a ketamine/xylazine mixture (90/10 mg/kg body weight). Blood samples were taken from the tail vein. A subgroup of mice received a subcritical calvarial defect of the size of 1.8 mm two weeks after induction of T1DM. After four weeks, the experiment was terminated (Fig. 1a).

All mice received a standard diet and drinking water *ad* libitum. Mice were separated in groups of 4 per cage and kept at room temperature in a 12 h light–dark-cycle. Each cage was enriched by a cardboard house. Approval for breeding the Dkk1^fl/fl^;Dmp1:Cre mouse line and for performing all invasive treatments was given by the institutional animal care committee of TU Dresden and the Landesdirektion Sachsen.

### RNA Isolation and quantitative PCR

Femoral bone was flushed and crushed in liquid nitrogen and the remains were immersed in Trifast (Peqlab, Germany). RNA isolation was performed according to the manufacturer's protocol. After reverse transcription using Superscript II (Invitrogen), cDNA was used for SYBR green-based real-time PCR. Primer sequences were as following: ß-actin s: GATCTGGCACCACACCTTCT, ß-actin as: GGGGTGTTGAAGGTCTCAAA; Dkk1 s: GCCTCCGATCATCAGACGGT, Dkk1 as: GCAGGTGTGGAGCCTAGAAG. Applied PCR conditions were: 2 min at 50 °C, 10 min at 95 °C continued by 40 cycles of 15 s at 95 °C and 1 min at 60 °C. A melting curve was created at 15 s of 90 °C, 1 min of 60 °C and 30 s of 95 °C. ΔΔCT method was used to calculate the results, which are presented as x-fold increase in comparison to beta-actin.

### Defect healing

A subcritical calvarial defect was created in mice under anesthesia (ketamine/xylazine mixture (100/10 mg/kg body weight) by intraperitoneal injection) by drilling a hole of 1.8 mm. Therefore, the head was shaved, an incision was made, and the calvaria was exposed. The defect was created with a carbide burr drill with a tip diameter of 1.8 mm and a rotation rate of 400 rpm (Fine Science Tools, Heidelberg) on both hemispheres. The defect was made carefully to avoid damage to the dura mater. After rinsing the defect thoroughly with saline, the skin was closed and disinfected to avoid infection. After the procedure, mice received metamizole in their drinking water (1.33 mg/ml) for 7 days to reduce pain.

### µCT and assessment of the calvarial defect

The bones were fixed in 4% PBS-buffered formaldehyde for 48 h and dehydrated with 50% EtOH. Bone samples were stored in 50% EtOH until further analysis. Bone mass and microarchitecture were determined by micro-computed tomography (μCT, vivaCT40, ScancoMedical, Switzerland) of femoral bone. The isotropic voxel size was 10.5 μm (70 kVp, 114 μA, 200 ms integration time). Femur scans included 100 metaphyseal slices, starting 1 mm proximal of the lower growth plate continuing upwards, and 150 diaphyseal slices, taken midpoint between the femoral head and femoral condylus. ScancoMedical protocols were used for all analyses. Measurements of trabecular bone mineral density (BMD) and the ratio of trabecular bone volume per total bone volume (BV/TV) were conducted on metaphyseal femur, whereas cortical BV/TV, cortical tissue mineral density (TMD), cross-sectional total area (TA), cross-sectional bone area (BA), and maximum and minimum area moments of inertia (Imax and Imin) were analyzed in diaphyseal slices. All µCT parameters adhere to international guidelines^25^.

The right halves of the calvariae were measured using the µCT with an isotropic voxel size of 10.5 μm, 70 kVp, 145 μA, and 300 ms integration time to generate three-dimensional reconstruction images of the defect areas. The area of newly formed bone at the bone defects was calculated from μCT-reconstructed images by using an ImageJ analysis system^26^.

### Bone histomorphometry

Calcein staining was performed to determine the bone formation rate in vivo. Therefore, mice received intraperitoneal injections of calcein (20 mg/kg, Sigma, Germany) 5 and 2 days before sacrifice. After fixation with 4% PBS-buffered paraformaldehyde tibias were dehydrated in an ethanol series with ascending concentrations. Methacrylate (Technovit 9100, Hereaus Kulzer, Germany) was used to embed the bones, which were then cut into 7 µm slices. Utilizing fluorescence microscopy, bone formation parameters such as the mineralized surface, mineral apposition rate, and bone formation rate were evaluated at the proximal tibia. To assess the number of osteoblasts, osteoclasts, and osteocytes, tartrate-resistant acid phosphatase (TRAP)-staining was performed on 2 µm thick, paraffin-embedded slices of the distal femur. Following international standards, Osteomeasure software (OsteoMetrics, Decatur, GA, USA) was used to obtain the just described histomorphometric data.

### Serum analysis

Blood was taken via heart puncture of anaesthetized mice and serum was collected after 15 min centrifugation at 400 g. The concentration of type 1 procollagen amino-terminal-propeptide (P1NP) as bone formation marker, C-terminal telopeptide (CTX) as bone resorption marker, Dickkopf-1 (Dkk1) and sclerostin were determined using ELISA following the manufacturer’s protocol (P1NP and CTX: Immundiagnostic Systems, Frankfurt am Main; Dkk1: R&D Systems, USA; Sclerostin: Alpco, USA). Serum samples used in sclerostin immunoassays were diluted 1:3. A mouse TRAP kit was used to determine the serum level of tartrate-resistant acid phosphatase 5b (TRACP 5b) according to the manufacturer’s protocol (Immundiagnostic Systems Limited, Boldon).

### Intraperitoneal glucose tolerance test

At an age of 16 weeks, right before sacrificing the mice, a glucose tolerance test was performed. Therefore, mice were fasted for 16 h and fasting blood glucose levels of mice were measured. Afterwards D-glucose (2 g/kg body weight) was administered via intraperitoneal injection and blood glucose levels were detected after 15, 30, 60, 120 and 180 min using a glucometer (ACCU CHEK Aviva III; Roche Diabetes Care, Mannheim, Germany). Blood samples were taken from the tail vein at indicated times.

### Statistical analysis

Results are shown as means ± standard deviation or as individual dots with the mean indicated as a horizontal line. Mouse cohorts were compared by calculating percentages based on mean differences within the genotypes. Two-way ANOVA with Bonferroni’s ad hoc tests were performed to evaluate statistical significance. P-values below 0.05 were considered statistically significant.

## Results

### Osteogenic Dkk1-deletion does not alter metabolic parameters in T1DM

Knock-down of Dkk1 in bone tissue was first validated by qPCR. Cre-positive animals in the control and the STZ-treated groups showed a strong reduction of Dkk1 mRNA expression (− 82% in non-diabetic Cre+ mice [p-value: 0.041], − 91% in Cre+ diabetic mice [p-value: 0.048], compared to their respective Cre− littermates). To determine if osteogenic deletion of Dkk1 impacts on the development of T1DM, all animals were weighed twice a week and blood glucose levels were measured. As shown in Fig. 1b, Dkk1-knockout in Dmp1-cre-expressing cells did not influence weight development of diabetic or normoglycemic mice. This was also reflected in the weight of subcutaneous and perigonadal fat pads, which was similar in Cre-positive and Cre-negative mice (Fig. 1c,d). Furthermore, Cre positivity did not protect from hyperglycemia, since twofold increased blood glucose levels were found in diabetic mice compared to non-diabetic controls in both genotypes (Fig. 1e). Moreover, all STZ-injected mice showed impaired glucose tolerance regardless of osteogenic Dkk1 expression (Fig. 1f). Thus, Dkk1 derived from late osteoblasts and osteocytes does not play a major role in T1DM pathophysiology.

### Lack of Dkk1 in osteoblasts and osteocytes mitigates T1DM-induced bone loss

**Figure 2:**
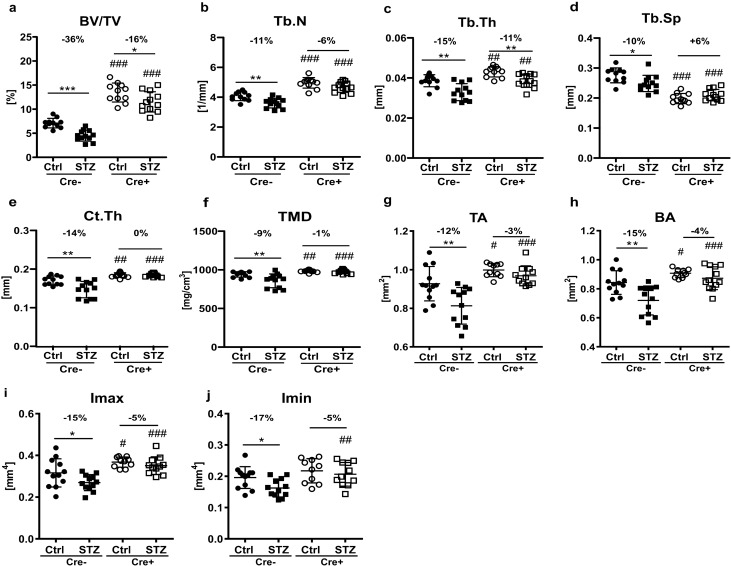
Deletion of Dkk1 in osteoblasts and osteocytes mitigates bone loss due to T1DM. (**a**) Trabecular bone volume per total volume (BV/TV), (**b**) trabecular number (Tb.N), (**c**) trabecular thickness (Tb.Th), and (**d**) trabecular separation (Tb.Sp) of the distal femur of male Dkk1;Dmp1-cre positive and negative mice with and without T1DM. (**e**) Cortical thickness (Ct.Th) of the femoral midshaft and (**f**) cortical tissue mineral density (TMD), (**g**) cross-sectional total area (TA), (**h**) cross sectional bone area (BA), (**i**) maximum moment of inertia (Imax), (**j**) minimum moment of inertia (Imin). Each dot indicates an individual mouse. N = 11–12 per group. Two-way ANOVA with Bonferroni post-hoc test. *p < 0.05, **p < 0.01, ***p < 0.001 vs. Ctrl; ^##^p < 0.01, ^###^p < 0.001 comparing Ctrl and STZ Cre− mice with their corresponding Cre+ littermates.

To investigate the degree of bone protection provided by knockdown of Dkk1 in osteoblasts and osteocytes in T1DM mice, we performed μCT analyses. All Dkk1 knockout mice showed a significantly higher baseline bone mass, while all diabetic mice showed a significant reduction of trabecular bone volume (Fig. 2a). The degree of bone loss however was mitigated in *Dkk1*^*fl/fl*^*;Dmp1:Cre*+ mice, showing only a 16% loss of bone volume as compared to -36% in Cre− mice (Fig. 2a). Furthermore, Cre− diabetic mice displayed a decrease in trabecular number, thickness, and separation compared to Cre− controls, whereas no significant or lesser distinct differences within these parameters were observed between the normoglycemic and diabetic Cre+ groups (Fig. 2b–d). Regarding cortical thickness of the femoral midshaft, Cre− diabetic mice showed a significant 14% reduction, however, no bone loss was detected in diabetic mice with osteoblast/osteocyte-specific Dkk1-deletion (Fig. 2e). Similarly, Cre− diabetic mice lost 9% of cortical TMD compared to their normoglycemic controls, while no change occurred in Cre+ diabetic mice (Fig. 2f). Both, cross-sectional total area and cross-sectional bone area, as well as the maximum and minimum moment of inertia (Imax and Imin), decreased significantly in wildtype diabetic mice compared to their buffer-injected controls, whereas no difference could be established between healthy and diabetic Cre+ mice (Fig. 2g–j). Also, Cre+ mice showed higher baseline values for the aforementioned parameters (Fig. 2g–j). Thus, these data demonstrate the potential of osteogenic Dkk1 knockout to mitigate trabecular bone loss and to completely abolish reduction of cortical bone in T1DM in mice.

### Dkk1 deletion in osteogenic cells does not protect from T1DM-induced inhibition of bone formation

**Figure 3:**
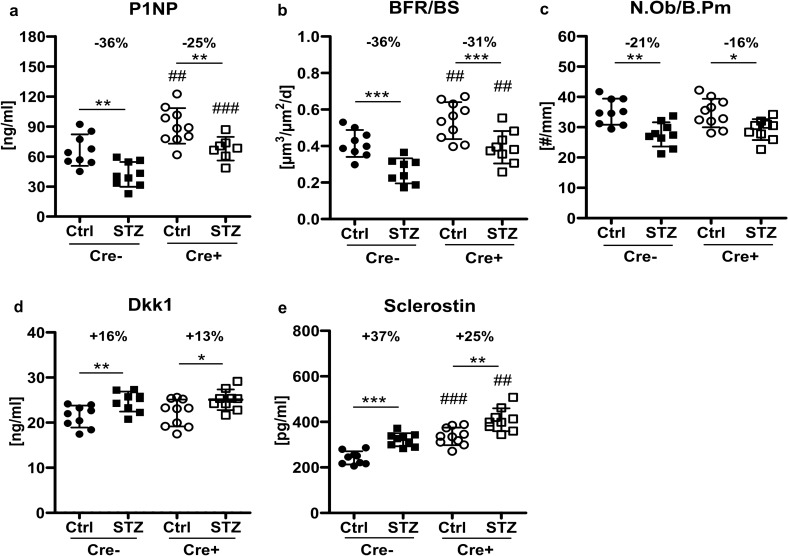
T1DM mediated loss of bone formation is not prevented by osteogenic Dkk1 deletion. (**a**) Procollagen type 1 amino-terminal propeptide (P1NP) serum level was measured by ELISA. (**b**) The bone formation rate per bone surface (BFR/BS) at the tibia was assessed histomorphometrically using calcein labels. (**c**) The number of osteoblasts per bone perimeter (N.Ob./B.pm) was quantified after tartrate resistant acid phosphatase staining at the femur. Quantifications were performed with Osteomeasure software. (**d**) Dickkopf-1 (Dkk1) and (**e**) sclerostin serum levels measured with ELISAs. Each dot indicates an individual mouse. N = 9–10 per group. Two-way ANOVA with Bonferroni post-hoc test. *p < 0.05, **p < 0.01 vs. Ctrl; ^#^p < 0.05, ^##^p < 0.01, ^###^p < 0.001 comparing Ctrl and STZ Cre− mice with their corresponding Cre+ littermates.

To investigate the mechanisms why bone loss was mitigated in Dkk1 conditional knockout mice, we further analyzed bone formation parameters using histomorphometry and serum bone turnover markers. All mice lacking Dkk1 in late osteoblasts/osteocytes displayed elevated P1NP levels and a higher bone formation rate than their wildtype littermates (Fig. 3a–c). However, these parameters as well as the number of osteoblasts were decreased to an almost similar extent in both diabetic groups (Fig. 3a–c). Blood levels of the Wnt inhibitors Dkk1 and sclerostin were increased in both diabetic groups (Fig. 3d,e), which may explain why osteogenic Dkk1 deficiency failed to prevent the inhibition of bone formation in T1DM.

### Knockout of Dkk1 in Dmp1-expressing cells ameliorates the T1DM-mediated increase in bone resorption

**Figure 4:**
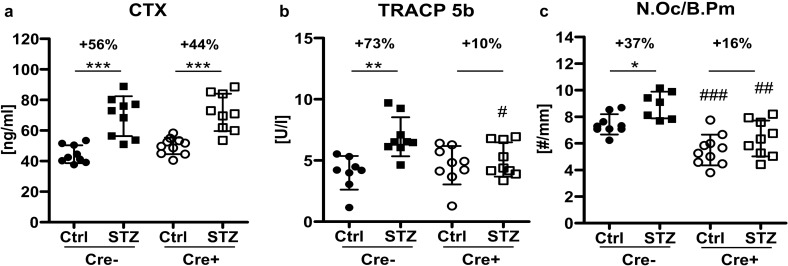
Lack of Dkk1 in osteoblasts and osteocytes ameliorates bone resorption in T1DM mice. (**a**) Serum carboxy-terminal collagen crosslinks (CTX) and (**b**) tartrate-resistant acid phosphatase 5b (TRACP 5b) measurements were performed using ELISA. (**c**) Trap staining was utilized to determine the number of osteoclasts per bone perimeter (N.Oc/B.Pm). Quantifications were performed with Osteomeasure software. Each dot indicates an individual mouse. N = 8–10 per group. Two-way ANOVA with Bonferroni post-hoc test. *p < 0.05, **p < 0.01 vs. Ctrl; ^#^p < 0.05, ^##^p < 0.01 comparing Ctrl and STZ Cre− mice with their corresponding Cre+ littermates.

In contrast to bone formation parameters, CTX as bone resorption marker, showed significantly increased serum levels in STZ-treated mice of both genotypes (Fig. 4a). Furthermore, STZ treatment increased serum levels of TRACP 5b in Cre− mice, while no difference was observed in Cre+ mice (Fig. 4b). Similar results were obtained at histological level, with Cre− controls showing 35% more osteoclasts than Cre+ controls and STZ increasing the number of osteoclasts in Cre− mice significantly by 37%, whereas Cre+ mice showed no significant induction of osteoclasts (Fig. 4c). Thus, except for CTX, T1DM-induced changes in bone resorption parameters were mitigated by osteogenic Dkk1 deficiency.

### Impaired bone regeneration in T1DM is not mediated by Dkk1

**Figure 5:**
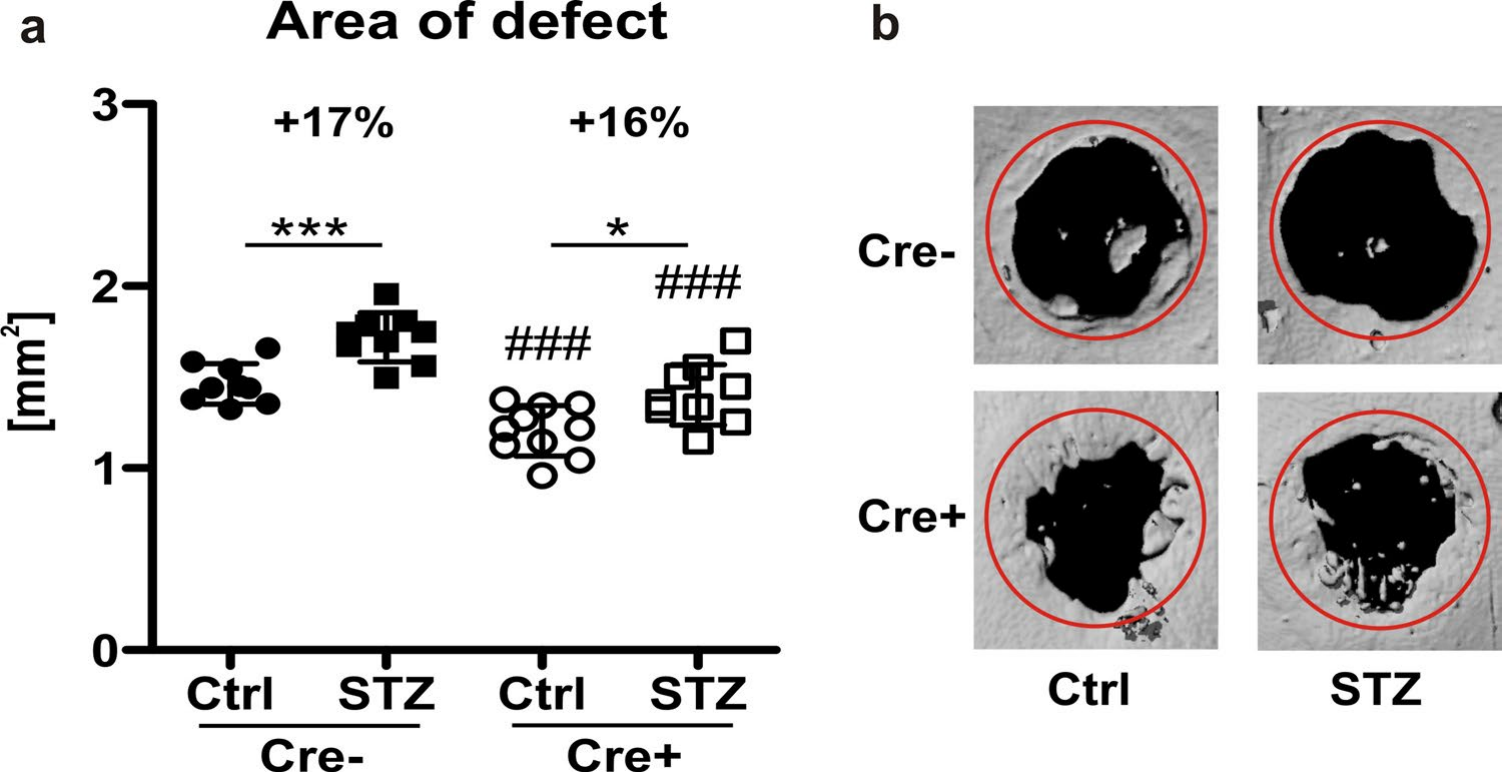
Bone defect healing in diabetic mice with an osteocyte-specific Dkk1-knockout. (**a**) Four weeks after a hole of 1.8 mm in diameter was drilled in parietal bone of male mice, the size of bone defects in mm^2^ was determined by µCT analyses. (**b**) Representative images of each group are depicted above. Each dot indicates an individual mouse. N = 8–10 per group. Two-way ANOVA with Bonferroni post-hoc test. *p < 0.05, **p < 0.01 vs. Ctrl; ^##^p < 0.01, ^###^p < 0.001 comparing Ctrl and STZ Cre− mice with their corresponding Cre+ littermates.

Finally, we assessed the impact of Dkk1 knockout in late osteoblasts/osteocytes on bone defect healing, as fracture healing is frequently impaired in patients with diabetes. The calvarial bone defect was used to obtain insights into the function of osteoblasts and osteoclasts during bone healing without confounding factors such as the callus and cartilage scaffold that are necessary for fracture healing. Therefore, a subcritical defect was created in the calvaria and healing was assessed after 4 weeks using µCT. The largest defect area was found in diabetic Cre− mice (Fig. 5a). This area was increased by 17% as compared to their normoglycemic controls. Cre+ control mice had the smallest defect area as seen in the representative μCT images (Fig. 5b) and as quantified in Fig. 5a. However, diabetic Cre+ mice showed similarly impaired bone defect healing as diabetic Cre− mice, with an increase in the defect area by 16%. In line with the low bone formation rates in diabetic Cre+ mice, it appears that defect healing cannot be rescued by deleting Dkk1 in osteogenic cells.

## Discussion

T1DM affects roughly 1.24 million people in the US^27^ and severely impairs bone strength by reducing bone formation and increasing bone resorption rates^28,29^. However, mechanistic details are not fully elucidated, but are required to develop targeted therapies to prevent bone loss and associated fractures. Previous studies indicate that Dkk1 serum levels are increased in children and adolescents with T1DM and also in skeletal tissue of mice with T1DM^19,20^. Thus, this paper aimed to clarify the bone protecting capacity of enhancing local Wnt signalling by knocking out Dkk1 in osteocytes and late osteoblasts.

Despite elevated levels of Dkk1 in the serum of T1DM mice, deletion of osteogenic Dkk1 did not affect weight development, fat distribution or glucose tolerance in diabetic mice. This is in line with our previous study in a high-fat diet model, in which lack of osteogenic Dkk1 also did not affect metabolic parameters^30^. Thus, these data suggest that the local production of Dkk1 by mature osteoblasts and osteocytes does not act in an endocrine way to affect glucose metabolism.

In contrast to glucose metabolism, deletion of osteogenic Dkk1 partially prevented T1DM-induced trabecular bone loss and completely prevented cortical bone loss. T1DM is known to reduce bone quantity and furthermore suppress the Wnt/β-catenin pathway^18,19,28,31,32^. Our results are consistent with another study postulating differential effects of β-catenin overexpression in mice with T1DM on trabecular and cortical bone^31^. Similar to our study, Wnt activation only ameliorated cortical, but not trabecular bone loss induced by T1DM, an effect the authors proposed to be dependent on differential Wnt16 expression.

Elucidating the underlying mechanisms of preserved bone mass, our data aligns with various publications showing bone formation is reduced in diabetic conditions in rodents^18,33,34^. Subsequently we investigated if osteogenic Dkk1 deletion, which is known to increase total bone volume and bone formation parameters^23,35^, could counteract these STZ-induced changes. Surprisingly, diabetic mice with osteogenic Dkk1 deficiency were not protected from decreased bone formation parameters, indicating the inability of local Dkk1 deletion to protect osteoblasts from impaired differentiation^36^ and increased apoptosis in T1DM^37^. This implies the possibility of systemic Dkk1 and/or sclerostin in suppressing bone formation in T1DM despite low levels of local Dkk1. Both parameters were increased in diabetic mice regardless of genotype. Currently, it is unknown which cell types contribute to the increased systemic levels of Dkk1 in T1DM, even in mice with reduced Dkk1 expression in osteogenic cells. Adipocytes in the bone marrow may constitute a source of Dkk1, as they have been shown to express Dkk1 and their numbers increase in T1DM, thereby further altering bone homeostasis and providing a proinflammatory environment^38,39^. As for sclerostin, levels were also elevated by osteogenic Dkk1-knockout under steady state conditions, leading to peak levels within the diabetic knockout group. In sum this supports current literature stating Dkk1 and sclerostin are elevated in type 1 diabetic individuals and further provides evidence for the intertwined compensatory circuits of the two Wnt inhibitors^20,23,36,40^. Accordingly, an approach blocking Dkk1 as well as sclerostin might be the most effective strategy to counteract bone loss in cases of severe osteoporosis.

Our key finding revolves around the osteoblast-mediated inhibition of bone resorption in diabetic Cre+ mice, which seems to be the most essential contributor to bone protection. While various studies suggest low or normal bone resorption rates in individuals with T1DM^18,41–45^, in the STZ mouse model, most studies report increased bone resorption rates^46–49^. Further, Coe et al. showed attenuated bone loss in diabetic mice under alendronate treatment^50^. The topic remains controversial, as they highlight the capacity of bisphosphonates to prevent osteoblast and osteocyte apoptosis rather than the suppression of osteoclasts^50,51^. However, also increased osteoclast parameters were reported to be at least partly responsible for reduced bone mass in T1DM^52,53^. Coherently with the latter, STZ induced an elevation in osteoclast activity and osteoclast numbers in Cre− mice, indicating a major role of increased bone resorption in diabetic bone loss. However, Cre+ mice were largely protected from this effect. Interestingly, serum CTX did not reflect the histological increase in osteoclast numbers, similar to our findings in healthy Dkk1-deficient mice, suggesting that Dkk1 rather affects osteoclast numbers than osteoclast activity^23,54^. Inhibition of Dkk1 has already been associated with suppressed bone resorption. Antibody-mediated Dkk1 inhibition resulted in decreased osteoclast formation in mice with arthritis and multiple myeloma^55,56^. This was mainly due to increased levels of the Wnt target osteoprotegerin in osteoblasts, which subsequently suppress osteoclastogenesis. Similar decreases of RANKL/OPG and osteoclastogenesis were found in Dkk1-deficient mice during steady state^23^ and after Dkk1 antisense treatment in ovariectomized mice^57^. Also, in this study, osteogenic Dkk1 deletion was sufficient to suppress the T1DM-induced increase in osteoclast numbers highlighting the importance of Dkk1 in regulating osteoclast rather than osteoblast function.

Finally, as T1DM is known to impair bone defect healing^58,59^, we investigated the role of Dkk1 therein. Diabetes significantly impaired bone defect healing in both Dkk1-deficient and wildtype mice, suggesting that Dkk1 does not play a prominent role in bone defect healing. As bone defect healing relies on active bone formation, yet, osteogenic Dkk1 deficiency was not able to overcome the T1DM-induced suppression of bone formation at the spine or tibia, we believe that this explains why bone defect healing was not improved by lack of Dkk1. However, it should be noted that blocking Dkk1 with antibodies improved tibial and femoral fracture healing in mice due to activated β-catenin signalling^40,60,61^. One major difference is that we created a bone defect in the calvarial bone, whereas the others investigated long bones, which underlie different ossification processes (intramembranous vs. endochondral ossification). Further studies are necessary to untangle these discrepant results.

Our study has limitations. This includes the model chosen to mimic T1DM. Although the low-dose treatment regimen with STZ is a common approach, the effect of STZ is enormously dependent on the dose, time and route of injection^32^. In addition, due to the fact that female mice do not develop overt T1DM after STZ injection, we only investigated the effects of T1DM in male mice. Finally, based on this study, it seems plausible that treatment of Dkk1 blocking strategies may improve bone mass in patients with T1DM. However, due to its lack of effect on enhancing bone formation under diabetic conditions, likely due to the compensatory increase in sclerostin, it may be more effective to block both Wnt inhibitors simultaneously.

In sum, this study shows that Dkk1 from Dmp1-expressing cells contributes to T1DM-induced bone loss mainly by increasing bone resorption. This mechanism appears to be more critical in cortical than trabecular bone, and more crucial for bone remodelling than calvarial bone defect healing.

## Data Availability

No large datasets were generated or analysed during this study. All other datasets analysed during this study are available from the corresponding author on reasonable request.
